# The Present Position of Psychotherapy
1A paper read at a Meeting of the Bristol Medico-Chirurgical Society on Wednesday, January 12th, 1921.


**Published:** 1921-06

**Authors:** R. G. Gordon


### THE PRESENT POSITION OF PSYCHOTHERAPY.[1]

BY

R. G. Gordon, M.D., B.Sc., M.R.C.P. Ed.

Psychotherapy has been very much in the public eye during the last few years, and has occupied a good deal of space not only in the medical press but also, unfortunately, in the lay press of the country. It has enjoyed a wave of popularity such as frequently sweeps over medical ideas, not always to the benefit of true scientific inquiry or even to the just appreciation of the subject which has temporarily become so popular. Appendicectomy, colectomy and vaccine therapy have been striking examples in recent years, and to-morrow we shall doubtless see vitamines occupying a similar position. After such waves there is inevitably a reaction, with the result that many really valuable observations may be lost sight of. With regard to psychotherapy, the time has undoubtedly come for everyone to make up his mind what the real value of this method of treatment is and what are its limitations. The first thing to notice is that psychotherapy is nothing new. Every good physician since

[1] A paper read at a Meeting of the Bristol Medico-Chirurgical Society on Wednesday, January 12th, 1921.

Hippocrates has practised this art in some form or other whenever he has considered his patient as a human being and not as a list of physical signs or laboratory experiments.

In the last fifty years, however, since Charcot first drew attention to the true nature of the neuroses, psychotherapy has been gradually evolved into a more definite scientific entity. Since 1914 the large numbers of neuroses dependent on the war has given us a field of study so productive that an enormous amount of progress has been made in estimating the value of the various theories previously held and in extending the scope of psychotherapeutic treatment. The three classical branches of psychotherapy are suggestion, persuasion, and analytical treatment. In the past there has been a great tendency to separate these one from another, and practitioners have devoted themselves to one particular branch to the exclusion of the other two. Most people are now satisfied that all are necessary in their therapeutic armament.

Suggestion has been practised consciously or unconsciously ever since medicine first became a study. The so-called miraculous cures of the Middle Ages were undoubtedly the result of suggestive measures, and to-day we still have these apparent miracles with us, though we now know that they depend on a perfectly normal attribute of the human mind. When we say that a person accepts a suggestion we mean that he accepts a proposition with conviction, without any rational grounds for this belief, and frequently in spite of the presence of adequate and rational grounds for its disbelief. In this process we must recognise two factors, firstly the suggestion which is offered and secondly the suggestibility of the patient. The suggestion may come to the patient from himself or from his environment. Suggestibility is a normal characteristic of mankind which may be increased by certain abnormal factors. The suggestion may be harmful or beneficial to the patient, and may result in the establishment of a symptom or in its removal, and in studying either the establishment or removal of such symptoms we must always consider both of the two factors mentioned above. The stronger the suggestion the less suggestibility in the patient need be looked for, and *vice versa*. If we construct two scales, one representing suggestibility and the other the strength of the suggestion, and place them inversely, we find patients developing symptoms at all points in such scales. A very strong suggestion will establish a symptom in a patient whose suggestibility is not much greater than that of the normal individual. For example, the actual presence of a disability in the patient's own person very readily suggests its continuance if no counter suggestion is brought to bear. When an injury has bruised but not divided any of the peripheral nerves a temporary paralysis is induced, but in course of time, when the nerve recovers from the bruise, potential power is restored to the limb. If, however, no suggestion has come to the patient that he can move his limb he will continue to accept the belief that it is paralysed, and he will continue to keep it immovable. Such cases have been of very great frequency after surgical injuries during the war, and treatment by massage and electricity, by tendon division or transplantation, have simply served to intensify the patient's conviction of the seriousness of his injury without doing anything to relieve it. On the other hand, it has been a very common experience that a few minutes' persuasion and counter suggestion have been sufficient to restore the function to a limb which has been paralysed for years, in spite of continual electrical or massage treatment. At the other end of the scale we have men who are so pathologically suggestible that the least thing will cause the establishment of a symptom. In such cases examinations by the physician, emotional shock, imitation from other patients are all that is required to produce the most extensive paralysis and disorganisation of function. Between these two extremes all varieties of cases can be found.

It is primarily to the credit of the French that it was discovered that such symptoms may be relieved by counter suggestion, whether by means of hypnosis or in the waking state, and frequently most dramatic recoveries have resulted from this process. Hypnosis is a condition analogous to sleep, in which a patient is temporarily withdrawn from the control of his rational and critical processes. In this state suggestions which do not run counter to his innate beliefs and tendencies are readily accepted, and hysterical symptoms can be induced or removed with ease. Similar results may be obtained without hypnosis by suggestive operations, electric shocks, baths and the like. It is often found that recovery from a symptom which has taken place under hypnosis is not lasting. For example, a patient who had had a contracted hand after a wound to his fingers for four years had been frequently hypnotised, with the result that his hand relaxed during this process but immediately contracted again on his being awakened, but ultimately the symptom was easily removed by persuasion. For this reason I consider that a method of treatment which allows the patient full insight into what is being done for him, and affords him an explanation as to why his disability originated and why it can now be removed, is much superior.

Another criticism of suggestive treatment is that if used alone it does not take into consideration the question as to whether the patient is pathologically suggestible or not. With this question I will deal presently.

The method of treatment whereby the patient is made to understand the whole process of the origin and of the cure of his disability may be described as persuasion, and for the removal of hysterical symptoms dependent on suggestion I consider it is far the best except in one condition, namely amnesia. In this case hypnosis is undoubtedly the method of choice, for memories can frequently be restored at once which would otherwise take weeks or months to elucidate.

In dealing with any case which is brought before one for the purpose of psychotherapy, it is necessary first of all to determine which of the symptoms are due to suggestion, and these symptoms I would describe as hysterical, following Hurst's definition. When these are found they should be removed as early as possible, and the ideal method for this purpose is as follows. The patient must be seen in a room by himself, and not amongst a crowd of others or in a noisy ward. He must be impressed that the whole object of the physician is to overcome his disability and to help him, and so far as possible he must be impressed with the physician's own belief, not only in the desirability of removing this symptom, but that it can be removed at once. When confidence has been established it must be explained to him how the symptoms originated and under what circumstances he accepted the suggestion. He must be persuaded that in spite of what all others have said to him his trouble does not depend on structural defect but on his own mental attitude. He must be shown how this particular function which he lacks has been forgotten by the rest of his personality, and he must be impressed with the importance of relearning how to control this function. Then with constant encouragement he is persuaded to make attempts to exercise the lost function, whether it be that of sight, hearing, movement or anything else, and the ideal which the physician must set before himself is that he shall not leave him until the restoration of the lost function is perfect. Often this can be achieved in a relatively short time, twenty minutes, three quarters of an hour or so, but sometimes it may take three or four hours. It is not always possible to give this time to one patient, but if it can be done it is time well spent, for if once the patient is perfect he only needs very slight supervision to see that he does not drop back into his old habitual ways. However, when the symptom is removed it must be clearly understood that the physician has not finished his work. He has not yet determined whether the suggestibility of the patient was normal or not, and four to six weeks of observation is necessary to see whether an anxiety neurosis may not have been underlying the hysterical symptom. This is usually the case in civilian cases, but by no means always so in war cases. In these the anxiety condition was frequently so slight that the removal of the hysterical symptom was all that was required to cure the patient.

If it is found that the patient is abnormally suggestible after removal of his hysterical symptom, or if he presents the symptom of an anxiety neurosis, the only treatment that in my opinion is of any use is some form of analysis. Under the heading of anxiety neuroses I propose to include all those conditions which are usually described as neurasthenia, and also the somnambulistic states on the one hand and psychasthenia on the other. The reason why I do this is that I consider all these conditions to be primarily due to the occurrence of mental conflict, and the treatment consists in the abolition of this conflict.

The term neurasthenia would be much better confined to the condition to which it was originally applied by Braid, namely the real exhaustion of nerve cells. This is not common, but does occur after really excessive physical work, such as long and arduous retreats in war time or after debilitating disease. It is in these cases that the Weir

Mitchell treatment does good ; but recovery ought certainly to take place as the result of three or four weeks' rest, and if the patient is no better within this time the diagnosis must be revised, and organic disease being excluded, some form of anxiety state will certainly be found to be present.

The knowledge gained in dealing with the anxiety neuroses depends on the work of the schools of Vienna and Zurich, and in order to understand their pathogenesis and the method of treatment it is necessary to consider briefly the views of these schools. Patients suffering from this trouble always present a certain degree of anxiety accompanied by its physical manifestations which the French call angoisse. This anxiety does not depend on anything very definite, and the patient is at a loss to explain why he should experience this unpleasant emotion. He will at the same time complain of insomnia, bad dreams, loss of power of recall and poor concentration. In more severe cases he will describe definite obsessions, whether these take the form of phobias, manias or of obsessive ideas ; or on the other hand he may partially lose consciousness of what he is doing and go through various pantomimes, to which no apparent meaning can be attached. Such conditions are described as somnambulisms, and vary in complexity from mild states of mental aberration to the full-fledged double personality.

Until Freud commenced to study the neuroses no adequate explanation had ever been given for these symptoms, and they seemed so nonsensical and absurd that the patients were generally dismissed as too childish and silly for the serious consideration of the physician. Freud pointed out, however, that there were many every-day activities, prejudices and mannerisms of the normal individual which were almost equally inconsequent and inexplicable. Being a determinist, he concluded that this could not be the result of mere chance, but must be the result of certain influences on our behaviour of which we are not conscious, and the part of the mind which was involved in the production of these mannerisms and also of the symptoms of the neuroses he termed unconscious.

Further investigation has shown that this unconscious part of the mind is made up of material derived from two sources. Firstly, there is the predisposition of the individual or the inborn tendencies which in large measure correspond to the instincts of the animals, such as the tendencies to fear, anger, disgust, and the impulses to action which depend on these emotions. Secondly, there are those events which happen to us and our reactions to them which we have considered too difficult or unpleasant to attach to our personality, and have therefore pushed away and refused to admit as applying to ourselves.

Such experiences, if sufficiently emotional and sufficiently concerned with our own activities, may not only be pushed out of active consciousness but also be almost completely divorced from our ego, and consequently may remain in the unconscious part of our mind, from where they exert their influence, although we are no longer aware of their existence. Just in so far as this unconscious material is moulded and welded together into a harmonious whole and not disturbed by discarded experiences which are too strongly emotional so will the individual be stable and of strong character, but just in so far as this material is inharmonious and conflicting so will the individual be neurotic.

Inasmuch as none of us reach absolute perfection in the structural harmony of our minds, we all occasionally act as the neurotic does, and indeed when we examine the latter's behaviour we find that it differs rather in degree than in quality from that of the normal individual. When such a lack of harmony exists a mental state is produced which is essentially disagreeable to the patient, so that he does his best to throw the whole of the incident which is producing the disharmony out of consciousness by the process which Freud has called repression. Further, the patient prevents its recurrence to consciousness by setting up a barrier which refuses it admission. This has been termed the censor. The influence of this repressed material on the individual life and behaviour depends, as has been said, on its intimacy with the patient's personality and the intensity of emotion which is attached to it. Incidents of low emotional intensity are repressed every day and cause no trouble, but those which exert enough influence on the personality to make completely successful repression impossible are the factors which underlie the neuroses. Such imperfectly repressed material acts as a dynamic foreign body in the consciousness of the patient, and consequently it tends to be manifested through the barrier of the censor, either directly or in symbolic manifestations of itself, in the form of the various symptoms of the psycho-neuroses, and it is not difficult to show how each symptom will depend on unsuccessful repression of a conflict. The treatment of the anxiety neuroses depends on the discovery of the repressed material and in harmonising it with the personality of the patient, a process which will vary in difficulty with the nature of the repressed material and the ability of the patient to adapt himself to circumstances. For this purpose Freud introduced the process which he called psycho-analysis.

Just as the symptoms of the psycho-neuroses are more or less symbolical expressions of unconscious material, so Freud found that this material also found expression more or less directly in the individual's dreams and in his mannerisms and eccentricities of behaviour. Hence we have three avenues of approach for investigating the unconscious activities in the patient's mind, some of which are supposed to be in conflict with each other. Firstly, we have the circumstances of these symptoms, how they arose, what they seemed to be dependable upon, what these factors in turn depended upon and so on, until they are traced to their primary cause; secondly, the observation of small mannerisms which might indicate what sort of tendencies there were and how they were making themselves felt; and thirdly a study of an analysis of dreams, which according to Freud is the royal road to the unconscious and consequently to the discovery of the origin of the mental disharmony. The process whereby the required information is usually obtained from these sources is called free association. Starting from the various elements of the dream, from a symptom, or a mannerism the patient is encouraged to allow his ideas to course through his mind without criticism and to relate them to the physician without withholding anything. By this method dynamic unconscious material will sooner or later make its appearance, and the basis of the conflict may be discovered. Other more technical methods, such as word association, are often of use in difficult cases. So far most psychotherapists will find themselves in agreement, and there seems to be very little doubt as to the correctness of the views so far enunciated.

It must be remembered that although these conceptions of the mental life of the individual may not have originated with Freud, yet he was the first to really formulate them in a definite theory, which had as its pragmatic object the treatment of the neuroses. For this work he deserves his due meed of praise, and will no doubt get it from posterity. It is, however, in the detailed application of the general theory of mental conflict that we find the differences which have split the psychotherapeutic schools so far asunder and have led to the disruptions which have taken place.

The Freudian school holds that the repressed conflict is always between the sexual tendencies of the patient and his environment, and therefore both the symptoms of the neuroses and the incidents in the person's dreams are necessarily sexual symbols. There has been more ink spilt and more heart-burnings endured over this word sex than over anything else in psychotherapy. This is very largely due to the fact that his critics, and even his followers, have forgotten the sense in which Freud used the word sex. We are too apt to consider sex on the purely appetite level, and forget that it has aspects not only on this level but also on the emotional and intellectual level. We all recognise that a woman's feelings towards life are very different from a man's, also that the average woman has a different intellectual outlook upon her environment to that of the average man. As an example of the common restriction of meaning of the term sex we have the extraordinary ideas relating to homosexuality. If anyone ventures to say that an individual has homosexual tendencies everyone jumps to the conclusion that it is meant that the unfortunate patient is guilty of grossly nefarious practices on the appetite level, whereas all that may really be implied is that the patient's emotional or intellectual attitude is that which is usually associated with the opposite sex, so that in his relationship with his own sex on these levels he takes up abnormal standpoints. That this confusion should exist among Freud's critics is reprehensible but perhaps natural, but that it should exist amongst his followers is perverse.

The second point that the Freudian school lays stress on is that the conflict was always started by some incident which usually took place in very early childhood and was then repressed. They go further than this, and say that the explanation of the amnesia for incidents in the first few years of life is due to the fact that many of these cases were of a perverse sexual nature and consequently actively repressed. This seems rather far-fetched, for surely the more simple explanation is that the young child has not got sufficient apperception-mass to which he may attach his memories.

The other point of the Freudian school is that treatment consists in discovering this real incident, and that once it is laid bare the patient is or should be cured. Experience has shown that this is by no means always the case.

Jung of Zurich diverged from this teaching in certain important respects. He denied that the conflict is always between the individual tendencies and the environment, but he considers that conflict may arise between the different tendencies themselves within the patient's own mind. Further, that the sexual tendencies were by no means always involved, though they might be and certainly were involved in a large number of cases. He admits that early experiences might be responsible for rendering the patient unadaptable to his environment, but that the onset of any given neurosis depended primarily not on the incident in the past but on something in the present which the patient could not face and which he was endeavouring to shirk. As a result of this shirking he tried to withdraw himself from reality and regress to the attitude towards life characteristic of an early stage of existence; for example, when an adult instead of accepting responsibility and acting for himself regresses to the infantile habit of mind and expects others to make up his mind for him and take on his responsibilities. The extension of this theory of regression which underlies Jung's theory of the correspondence between individual dreams and racial mythology, though a subject of intense interest, is too complicated to enter into here. Lastly, Jung laid it down that treatment did not simply consist in laying bare the conflict and in showing the patient that he was not always adapting himself to life and that he was shirking his difficulties, but that the physician was responsible for the reconstruction of the patient's attitude, and he must see to it before he leaves him that he is once more enabled to adapt to life as it is.

Adler of Vienna announced a theory of the utmost importance, which one frequently finds applicable in the cases with which one is called upon to deal. He maintained that the neuroses were due to a conflict between a consciousness of inferiority which the patient refuses to admit and what he calls the will to power, which is a mechanism whereby the patient strives to prove to himself the absence or unimportance of his physical abnormality.

Such, then, is a very brief description of the tendencies of the continental schools of psycho-analysis. A school of psychotherapists is growing up in this country whose views were admirably stated by Ross at Cambridge last June. They refuse allegiance to any one continental school, though they find themselves in closest approximation to Jung. They strive to take the valuable elements from each and try to avoid their excrescences. While admitting that sex is often the basis of conflict, they consider the insistence upon the universality of sexual conflict is very misleading and tends to absurdity. As, for example, the statement of Ferenczi that he has traced many neuroses to the fear of the fœtus at passing through the maternal passages. They consider that every case must be considered on its own merits as to the conflicts involved and the symbolism employed by the individual's consciousness. While recognising that every individual thinks symbolically, they regard the insistence that certain incidents and pictures in dreams always have the same symbolic meaning as dangerous or even ridiculous: for example, the statements that all dreams concerning Zeppelins have necessarily a phallic insigficance.

A good example of the importance of not insisting on a general symbolism in individual cases can be given from my own experience. A man had a dream about snakes. Admittedly the significance of snakes is universally phallic in folk-lore, but in this man's case it referred to a particular emotional experience during the war in which a large snake had come over a wall under which he was standing and had slid down over his shoulder. It was, in fact, an ordinary war dream.

They further consider that the insistence that dreams are necessarily expressions of positive conations or wishes is erroneous, as they may just as well be expressions of negative conations or fears. While admitting the necessity in a few cases of the extended and elaborate analysis preached by the Freudian school, they believe that as a rule care should be exercised to keep the analysis within bounds and only try to discover the conflicts actually causing the neuroses, as the enormous amount of material elucidated by the former methods tends only to mystify the patient and the physician. Again, while the elaborate processes of dream analysis and word association tests may be of great help in certain difficult cases they are not necessarily required as a routine measure. They also hold that the physician has not completed his duty to his patient until he has established him in a successful occupation in such a way that his self-confidence and self-respect are restored. Further, they do not believe that psychotherapy as a whole and analytical methods in particular are or can be universally successful, and recognise that the chief barriers to success are the lack of sympathy, open-mindedness and patience on the part of the physician, lack of intelligence or the desire of recovery on the part of the patient, or his complete inability to face and adapt himself to the conflicts which underlie his neuroses.

In dealing with an anxiety neurosis, the object we have before us is to define the mental conflict which is causing the trouble and to make the patient face it and realise its actual value, and to help him to find some solution whereby it will be resolved. To do this we have first of all got to take a careful history of all that the patient can remember about the onset and progress of his disease, and to investigate more or less fully his general behaviour, and determine what sort of mental type a patient is. From our knowledge of psychology we may then know the sort of way in which he will act to given circumstances in his environment. From gaps and peculiarities in his story we can get some indication of repression and incidents which have been banished to the unconscious mind. In the progress of our talks with him we notice his mannerisms, his prejudices and his eccentricities. We ask him to tell us about any dreams he can remember, and I do not consider it is necessary or desirable to try and analyse and find the meaning for each one, for by careful attention to them we can often get a great deal of help, and very frequently one particular dream will give a key to the whole process.

It would be impossible in a short paper such as this to attempt to give any outline of the details of analytical treatment, but perhaps an example will show the sort of thing which is achieved.

A lady suffering from many neurotic symptoms was generally regarded as very hysterical, and had an intense phobia of all illness. This dread of illness was found to go back to an occasion when she was anæsthetised for the purposes of examination. It was found that she had no objection to the examination, but that it was the first time when the thought had occurred to her that she might die. This raised the problem as to why she was so abnormally frightened of dying, and from her emotional reactions at the time it was obvious that death had a terror for her quite out of proportion to what is normally the case. After further investigation it was found that she had been brought up in early childhood on strict Calvinistic lines, and she had early attained great dread of the hereafter and the punishment it might involve if she did anything wrong. Being very musical and always intended for operatic work, she had been taken when about seven years old to see *Faust*. This was the first opera she had seen, and she was very much impressed by it. Soon after she broke a favourite vase of china belonging to her mother, of whom she was rather frightened. In terror lest she should be discovered she hid it in the dust-bin, but next day to her horror the dustman came to get this away while she and her mother were present. In her intense anxiety she resolved that if the china was not seen she would sell her soul to the devil. She was not discovered, but it quickly came to her mind what an appalling thing she had done. When this memory was brought up to her she went through a considerable emotional crisis and said, "Well, no wonder I am afraid to die or afraid of being ill. What shall I do?" She was still looking at things from her childish point of view. When, however, one had talked to her for a time she was able to see that a child of seven can hardly be held morally responsible for an action the meaning of which she did not really understand, and which had been undertaken in a moment of intense emotional stress. After this was cleared up her entire attitude towards life changed, and she was able to go about her work and attend to social duties in a way which had been impossible for years. She no longer imagined that every little pain meant instant death, and consequently her own happiness and that of those about her was improved to a very marked extent.

In conclusion, I would like to lay down the following points. So far as the psycho-neuroses are concerned some form of psychological treatment is always necessary, and purely material therapy, whether massage, hydrotherapy or drugs, if used without this will really do the patient more harm than good, though such measures may be advisable or even necessary if strictly subsidiary to psychotherapy.

All the various methods of psychotherapy are useful for removal of hysterical symptoms, but persuasion is the method of choice. In some cases this is sufficient in itself, but in other cases, where an anxiety condition underlies the symptom, this must be treated by some form of analysis.

In anxiety neuroses some degree of analytical treatment followed by reconstructive measures are necessary.

While hypnosis is useful in removing certain symptoms it is not satisfactory as a general therapeutic measure, because it does not give the patient sufficient insight into his own condition, and never explains all the whys and wherefores of his illness.

Psychotherapy, and especially psycho-analysis, is frequently grossly abused by practitioners who will not distinguish between functional and structural change, and thereby render themselves ridiculous, as for example did the exponent of the art who claimed with some pride at an important medical meeting to have cured a case of epistaxis by eighteen months' psycho-analysis.

The present-day tendency for the laity to practise psycho-analysis is dangerous and pernicious, and the practice of society ladies psycho-analysing their butlers as described in the *Daily Mail* is indecent. On the other hand, many practitioners are blind to the possibilities of psychotherapy, and outside the realm of the psycho-neuroses many conditions commonly regarded as essentially organic can be dealt with by this method. As examples I may mention the cases of pernicious vomiting of pregnancy described by Hurst, in one of which the ammonia coefficient reached 25. All these were ordered by the gynecologist to have the uterus emptied within twenty-four hours if life was to be saved, and all of them were cured by psychotherapy during that interval and have gone on to full time without further accident. Also two cases of deaf-mutism dating from early infancy who had been cured, one at the age of seventeen and the other at twenty-two.

In medical education the absence of any teaching of medical psychology is a serious deficiency. The course of lectures on mental diseases does not supply this want at all in most schools, and consequently the medical student enters into practice with no knowledge of how to deal with his neurotic patients, which form a very large proportion of his practice, and, what is almost more important, has no organised knowledge of how to approach the subjective symptoms he meets with, which as Sir James Mackenzie has said are really just as important as the objective signs.

